# Routine treatment pathways of patients with major depression and active suicidal ideation with intent in Italy: interim results from the ARIANNA observational study

**DOI:** 10.1192/j.eurpsy.2022.397

**Published:** 2022-09-01

**Authors:** M. Pompili, A. Bellomo, E. Pilotto, G. Rosso, M. Adami, D. Andreis, B. Roncari, D. Delmonte

**Affiliations:** 1 Sant’Andrea Hospital - Sapienza University of Rome, Department Of Neurosciences, Mental Health And Sensory Organs, Suicide Prevention Center, Rome, Italy; 2 University of Foggia, Department Of Clinical And Experimental Medicine, Psychiatric Unit, Foggia, Italy; 3 ULSS8 Berica - Vicenza Hospital, Department Of Mental Health, Spdc I, Vicenza, Italy; 4 San Luigi Gonzaga Hospital - University of Turin, Department Of Neuroscience “rita Levi Montalcini”, Turin, Italy; 5 Janssen-Cilag SpA, Department Of Medical Affairs - Neuroscience, Cologno Monzese, Italy; 6 MediNeos Observational Research - IQVIA, Data Management & Statistics, Modena, Italy; 7 MediNeos Observational Research - IQVIA, Clinical Operations, Modena, Italy

**Keywords:** suicidal ideation, major depressive disorder, real world, standard of care

## Abstract

**Introduction:**

Major depressive disorder (MDD), especially in case of suicidal risk, is a psychiatric emergency, associated with high patient burden and healthcare resource utilization. Although active and urgent treatment is crucial, little is known on comprehensive care management of this condition in Italy.

**Objectives:**

Here we report the ARIANNA study [NCT04463108] interim results to primarily describe the treatment utilization pathways of patients with MDD and active suicidal ideation with intent in the current clinical practice in Italy.

**Methods:**

This observational prospective cohort study included adult patients with a moderate-to-severe major depressive episode (MDE) and active suicidality from 24 Italian sites. Real-world data on patient characteristics, treatments, clinical outcomes, and healthcare utilization were collected during a 90-day follow-up. Data collection is still ongoing.

**Results:**

Sixty-four evaluable patients were considered for this interim analysis: 41 (64.1%) females, mean [SD] age 46.0 [15.4] years, a concomitant psychiatric diagnosis in 7 (10.9%), and other comorbidities in 26 (40.6%). The baseline mean [SD] MADRS total score was 37.5 [7.2], with severe MDE and prior suicidal behavior in 30 (46.9%) and 21 (32.8%) patients, respectively. Median [25th;75th percentiles] duration of current MDE was 1.1 [0.3;2.1] months. Acute inpatient hospitalization was provided for 43 (67.2%) patients. Antidepressant augmentation with mood stabilizers and/or antipsychotic drugs and optimization were the most frequent early standard-of-care treatment regimens in 32 (53.3%) and 24 (40.0%) patients with available data (N=60), respectively.

**Conclusions:**

Our preliminary results suggest that initial treatment approaches in this critical population are mostly polypharmacological and delivered as inpatient care, with consequent intensive resource utilization.

**Disclosure:**

The ARIANNA study was sponsored by Janssen-Cilag SpA, Italy. DD and MA are employees of Janssen-Cilag SpA. DA and BR are employees of MediNeos S.U.R.L., a company subject to the direction and coordination of IQVIA Solutions HQ Ltd.

